# Assessment of Pulmonary Vein Diameters in Cavalier King Charles Spaniels with Myxomatous Mitral Valve Disease

**DOI:** 10.3390/vetsci12070615

**Published:** 2025-06-24

**Authors:** Carlotta Ferri, Juliette Besso, Hugues Gaillot, Yannick Ruel, Albert Agoulon, Christophe Bourguignon, Clémence Mey, Vassiliki Gouni

**Affiliations:** 1 ADVETIA Veterinary Hospital, 9 Avenue Louis Breguet, 78140 Vélizy-Villacoublay, France; 2 Vetradmobile, 97 Rue Monge, 75005 Paris, France; 3 Oniris, INRAE, BIOEPAR, 101 Route de Gachet, 44300 Nantes, France; 4 Médi-Vet Veterinary Hospital, Avenue de Montoie 47, 1007 Lausanne, Switzerland

**Keywords:** canine, myxomatous mitral valve disease, pulmonary vein, Cavalier King Charles Spaniels, congestive heart failure

## Abstract

The present study aimed to compare pulmonary vein (PV) diameters between Cavalier King Charles Spaniels (CKCSs) with myxomatous mitral valve disease (MMVD) and healthy CKCSs, assess correlations between PV diameters and echocardiographic parameters, and identify the optimal PV diameter cut-off value that distinguishes stage C from stage B2. CKCSs were recruited both retrospectively and prospectively and classified according to the ACVIM guidelines (stages A, B1, B2, and C). From a left apical view, the diameters of three PVs (PV1, PV2, and PV3) were measured with high reproducibility. In healthy dogs, the PV2 diameter showed no correlation with body weight. The PV2 diameter was significantly higher in stage B2 compared to B1 and in stage C compared to B2, while no difference was found between stages A and B1. The median (IQR) PV2 diameters were 4.9 mm (3.9–5.2) in stage A, 5.1 mm (4.0–6.0) in stage B1, 9.3 mm (7.3–11.1) in stage B2, and 13.7 mm (9.9–15.1) in stage C. Positive correlations were observed between the PV2 diameter and the left ventricular internal diameter normalized for body weight, the left atrium-to-aorta ratio, mitral E wave peak velocity, tricuspid regurgitation pressure gradient, and regurgitant fraction. A PV2 diameter cut-off value of 12.8 mm discriminated stage C from stage B2 with 57% sensitivity and 93% specificity. The PV2 diameter is a reproducible echocardiographic measure that increases with MMVD severity and could assist in the early detection of congestive heart failure. However, the modest sensitivity observed reflects the overlap of PV2 measurements between stages B2 and C. Therefore, PV2 should be interpreted with caution and considered a supportive, rather than exclusive, tool in disease staging and therapeutic decision-making.

## Simple Summary

This study examined whether the size of the pulmonary veins could help assess heart disease severity in Cavalier King Charles Spaniels (CKCSs) with myxomatous mitral valve disease (MMVD). Healthy dogs were compared to those at different stages of the disease using ultrasound measurements. The pulmonary vein 2 (PV2) diameter was found to be the least affected by body weight and increased as the disease progressed. The PV2 diameter showed positive correlations with several echocardiographic indices of cardiac remodeling. A PV2 cut-off value of 12.8 mm yielded a sensitivity of 57% and a specificity of 93% for distinguishing stage C from stage B2. PV2 measurements could be used as an additional, not standalone, tool for staging and treatment decisions and may offer additional insights into cardiac disease progression.

## 1. Introduction

Myxomatous mitral valve disease (MMVD) is the most common cardiac condition in dogs, accounting for approximately 75% of all cases of canine heart disease [1]. Structural changes to both the cellular and extracellular components of the mitral valve leaflets, including progressive dyscollagenesis and the expansion of the proteoglycan-rich spongiosa layer, compromise valve coaptation [1,2,3]. Worsening mitral insufficiency leads to maladaptive neurohumoral activation, volume overload, eccentric hypertrophy of the left ventricle (LV), and left atrial dilation. Typically, these symptoms accompany the development of left-sided congestive heart failure (CHF) [1].

The progression of MMVD is reflected in the staging (A, B1, B2, C and D) provided by the American College of Veterinary Internal Medicine (ACVIM) consensus [1]. Stage A includes dogs at increased risk but without structural abnormalities [1]. Stage B (including B1 and B2) refers to dogs presenting with a left apical systolic heart murmur audible on auscultation, but without clinical signs. In stage B1, the dimensions of the left heart chambers are within normal limits or only mildly dilated, and they do not meet the criteria defined by the EPIC study [4]. Conversely, dogs in stage B2 exhibit enlargement of both the left atrium (LA) and LV, fulfilling the EPIC study criteria, specifically a left atrial dimension to the aortic valve annulus dimension (LA:Ao) in early diastole equal to or exceeding 1.6 and a left ventricular internal diameter normalized for body weight (LVIDDN) of at least 1.7 [1,4,5,6]. Stage C includes dogs with current or past clinical signs of heart failure (HF) that respond to standard therapy. Stage D encompasses dogs with refractory HF requiring advanced medical management [1].

Cardiogenic pulmonary edema occurs once severe mitral insufficiency has exceeded the capacitance of the usually dilated LA and pulmonary veins (PVs), elevating pulmonary capillary pressure above plasma oncotic pressure so that the accumulation of modified transudate within the pulmonary interstitium overwhelms lymphatic drainage. There is a clear link between the development of clinical signs of CHF, the increase in left atrial pressure and size, the dilation of PVs, and the occurrence of pulmonary edema, all of which can be exploited by imaging modalities to make a diagnosis [1,7,8].

Confirming a diagnosis of left-sided CHF can be clinically challenging. Patients may be presented in a clinically unstable state that necessitates rapid intervention before traditional forms of imaging such as chest radiography can be utilized or biomarker (N-terminal pro-B-type natriuretic peptide) results are available [1]. Echocardiography may not be readily available in general practice settings, and affected dogs may have concurrent and significant respiratory and mitral diseases [1]. Radiographic interpretation can also be influenced by thoracic conformation and breed variability, and definitive thresholds for staging such as the vertebral heart score (VHS) and the vertebral left atrial size (VLAS) pertain more to differentiating B2 from B1, rather than C from B2 [1]. Whatever the above-mentioned diagnostic limitations, the early treatment of dogs with CHF is essential. For dogs with MMVD and CHF, diuretics are the cornerstone of therapy to manage life-threatening clinical signs [1]. Identifying the optimal timing to treat with diuretics in the early stages of left-sided CHF remains challenging.

In people, recent studies showed that an increase in pulmonary vein (PV) diameter and left atrial size occurs in patients with left-sided CHF before the onset of clinical signs [9,10]. The PV diameter has also been identified as a useful echocardiographic parameter to initiate or tweak diuretic therapy and assess its impact on patients with CHF and preserved ejection fraction [10], thus limiting in-patients.

In dogs, as well as in cats and horses, the echocardiographic diameter of the PV and right pulmonary artery has been compared [11,12,13]. Healthy dogs have a pulmonary vein diameter-to-pulmonary artery diameter ratio (PV/PA) of approximately 1.0 [11]. The PV/PA ratio increases with the severity of HF in dogs with MMVD, allowing for the identification of dogs with CHF [14]. However, its predictive accuracy may be impacted: some respiratory conditions such as idiopathic pulmonary fibrosis and parasitic heart or lung diseases can cause precapillary pulmonary hypertension (PH), and severe left-sided HF can cause postcapillary PH, both resulting in pulmonary artery enlargement and a reduced PV/PA ratio, proportionate to PH severity [15,16,17,18].

In dogs, the PV anatomy is more complex than in people, typically consisting of 7 veins (from 5 to 8) [19]. Using two-dimensional echocardiography, the regions of termination of the pulmonary veins into the LA can be visualized as three major ostia resembling the four ostia commonly observed in people [19]. The three canine ostia include (i) the cranial left ostium, which receives the cranial and caudal veins of the left cranial lung lobe; (ii) the caudo-dorsal ostium, which receives the veins of the left caudal, the accessory, and the right caudal lung lobes (with some variation in the location of the accessory vein); and (iii) the right ostium, which receives the veins of the right middle and right cranial lung lobes [19,20]. These three ostia are well visible on a left parasternal long axis echocardiographic view.

The echocardiographic PV diameter has diagnostic and therapeutic relevance in people [10], but it has not yet been assessed in dogs. To narrow down normal values, we chose to focus on only one breed, the Cavalier King Charles Spaniel (CKCS), because of its high risk for developing MMVD [1,21]. We hypothesized that PV diameters would increase with MMVD severity, correlate with echocardiographic parameters, and allow discrimination between the clinical and preclinical stages. The aims of this study were therefore to (1) measure and compare PV diameters between dogs in different stages of the ACVIM classification of MMVD, (2) provide cut-off values for stage discrimination, and (3) correlate PV diameters to several clinical and Doppler echocardiographic parameters.

## 2. Materials and Methods

### 2.1. Animals

The study population included a diseased group of dogs with MMVD classified according to ACVIM consensus guidelines [1] and a control group.

The control group initially consisted of 37 CKCSs prospectively recruited at ADVETIA Veterinary Hospital between January and March 2023. The inclusion criteria for this group were clinically healthy dogs without heart murmurs and echocardiographic abnormalities of the mitral valve (stage A).

The diseased group included dogs enrolled retrospectively either at ADVETIA Veterinary Hospital or in a mobile consult setting for small animal practices (Vetradmobile, Paris area) between January 2022 and December 2022, as well as dogs enrolled prospectively at ADVETIA and at the Médi-Vet Veterinary Hospital from January 2023 to January 2024. Inclusion criteria were an echocardiographic diagnosis of MMVD associated with a left apical systolic heart murmur [22]. Dogs with concurrent systemic illnesses or congenital heart defects were excluded.

### 2.2. Echocardiography

Each dog had a transthoracic echocardiographic exam performed by either a board-certified cardiologist (VG and CB), a cardiology resident (CF), or a board-certified radiologist with more than 20 years of experience in echocardiography (JB). Equipment used were a Vivid E90 ultrasound system (VG, CB, CF; VE 90, General Electric Ultrasound system, Boston, MA USA) using 2.7–8 MHz and 2–4.5 MHz phased-array transducers and a Philips CX50 system (JB; Philips Healthcare, Suresnes, France) using an S5-1 MHz phased-array transducer. All standard measurements were acquired from 2D mode, M-mode, and conventional Doppler in standing dogs as previously described [23], with simultaneous single-lead electrocardiogram recordings.

Standard right parasternal (long and short axes) and left apical parasternal views were used for data acquisition. The left atrial size was assessed using the LA:Ao, which was measured on the right-sided short axis view in early ventricular diastole. The measurement was taken in the first frame after aortic ejection [5]. The LVIDDN was measured at the end of diastole in M-mode from a right parasternal short axis view of the left ventricle at the level of the chordae tendineae [6]. Mitral inflow was assessed using pulsed-wave Doppler from the left apical parasternal four-chamber view by measuring the peak velocity of early diastolic transmitral flow (E) and the peak velocity of late diastolic transmitral flow (A) and calculating the E-to-A ratio (E:A) [24]. Mitral regurgitation was assessed using the proximal isovelocity surface area (PISA) method, and the regurgitant fraction (RF) was calculated for dogs with one regurgitant jet [25]. The velocities of potential tricuspid regurgitation (TR) and the associated calculated transvalvular pressure gradient (PG) [26] were recorded.

### 2.3. Acquisition and Reproducibility of PV Diameters

**Figure 1:**
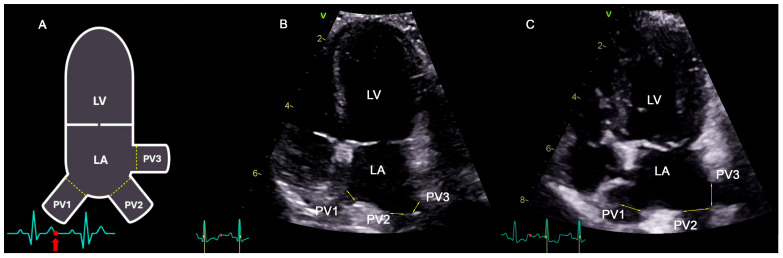
Schematic representation (**A**) and echocardiographic images of a control dog (**B**) and a stage C (**C**) dog from the left parasternal apical view focused on the ostia of the pulmonary veins. PV diameter measurement was performed on 2D echocardiography using the inner edge-to-inner edge method (indicated by the yellow dotted line in panel (**A**) and yellow double arrows in panels (**B**,**C**) at the end of systole, as indicated by the red arrow on the schematic electrocardiogram at the bottom of the image. Three PVs (PV1, PV2, and PV3) were identified, and their diameters were recorded. PV1, pulmonary vein 1; PV2, pulmonary vein 2; PV3, pulmonary vein 3; LA, left atrium; and LV, left ventricle. The V in panels (**B**,**C**) corresponds to the probe’s orientation marker.

Pulmonary vein diameters were measured in 2D mode from a left parasternal apical, long axis view, where the three major ostia, namely right ostium (PV1), caudo-dorsal ostium (PV2), and cranial left ostium (PV3), were clearly visualized (Figure 1). Measurements were taken using the inner edge-to-inner edge method during end-systole (end of the T-wave on ECG, maximum closure of the mitral valve on 2D images), as this phase represents the point of the maximum vein diameter [27]. All PV measurements were consensually taken by the cardiology resident (CF) and a board-certified cardiologist (VG).

To assess intra- and interobserver variability, bidimensional recordings from 10 randomly selected dogs from both the healthy and diseased groups were reviewed by two observers (CF and VG) independently and blindly. Measurements were repeated three times on the same day, during three non-consecutive days.

### 2.4. Thoracic Radiography

For each dog, right lateral and ventro-dorsal thoracic radiographic views were obtained at the time of the echocardiographic exam. In cases of respiratory distress, a dorsoventral view was used instead of the ventro-dorsal projection. The images were reviewed independently by two board-certified veterinary radiologists (YR and HG). The radiographic studies were in a random order, and the radiologists were blinded to the animals’ identity, examination date, and echocardiographic results. A scoring system evaluating cardiac size, lung pattern, and pulmonary venous diameter was used as previously described by Merveille et al. [14]. The scoring system was adapted to the specifics of our study, which was performed on a single breed.

The assessment of cardiac silhouette size was performed by summing 3 scores related to (1) a qualitative and semi-quantitative assessment of the cardiac silhouette size, which was performed on a lateral view based on the cranio-caudal length of the cardiac silhouette relative to the number of intercostal spaces, the angle between the thoracic trachea and the cranial thoracic spine, the contact between the cardiac silhouette and the sternum, the dorsal displacement of the trachea and carina, and the straightening of the caudo-dorsal aspect of the cardiac silhouette and on a ventro-dorsal view based on the width of the cardiac silhouette relative to the thoracic width and the bulging of the cardiac silhouette in the projection areas of the left atrial body and left atrial auricle (normal = 0 and enlarged = 1); (2) a measured VHS using published reference values for CKCSs [28], where VHS ≤ 10.64 = 0 and VHS > 10.64 = 1; and (3) a measured VLAS using reference values for CKCSs [28], where VLAS ≤ 2.09 = 0 and VLAS > 2.09 = 1. Lungs were assessed and given a score of 0 if normal, 1 for an interstitial pattern, and 2 for an alveolar pattern. Pulmonary vein diameters were evaluated on right lateral thoracic radiographs at the level of the fourth rib by comparing the diameter of the right cranial PV to that of the right cranial pulmonary artery. For ventro-dorsal views, the diameter of the caudal PVs was compared to the diameter of the accompanying pulmonary arteries. Congestion was scored as 0 when the vein diameter appeared within normal limits, 1 when a mild congestion was suspected, and 2 when venous dilation was obvious [29]. The sum of the 3 scores rating the cardiac size, lung pattern, and PV size is the radiographic score (ranging from 0 to 7) for each dog. Ratings from the two radiologists were subsequently compared.

### 2.5. Statistical Analysis

Statistical analysis was performed using R version 4.4.1 (https://www.Rproject.org/, accessed on 30 June 2024), with the significance level set at *p* < 0.05, except when a Bonferroni correction was applied (see below). Normality was tested using the Shapiro–Wilk test. Continuous data were expressed as medians and interquartile ranges (IQR: 25th–75th percentiles). Categorical data were expressed as ratios and percentages and were compared with Fisher’s exact test for proportions. Correlations between PV diameters and body weight (BW) or echocardiographic parameters were analyzed using Spearman’s rank correlation test. Differences in the distribution of the PV2 diameter and other echocardiographic continuous data among groups were evaluated using the Kruskal–Wallis rank sum test, followed by pairwise comparisons (6 combinations between stages A, B1, B2, and C) with the Wilcoxon rank sum test. For these pairwise comparisons, a Bonferroni correction was applied: the significance level was set to *p* < 0.008 (corresponding to 0.05/6 because 6 comparisons were performed).

The readers’ performance in distinguishing dogs with different stages of MMVD was tested for PV diameter by receiver operating characteristic (ROC) curve analysis with the package pROC. Areas under the ROC curves (AUCs) were calculated and expressed with the 95% confidence interval (CI). Values of AUCs of <0.7, 0.7–0.8, 0.8–0.9, and >0.9 were classified as poor, acceptable, excellent, and outstanding performance, respectively [30]. The optimal cut-point was selected using the package Optimal-Cutpoints by mean of the Younden index (sensitivity + specificity − 1). Sensitivity, specificity, and positive predictive values (PPV) were calculated considering the optimal cut-point.

Intraobserver and interobserver variability were calculated using the coefficient of variation (CV) of measurements on a sub-sample of 10 dogs randomly chosen and blindly measured by the two observers (CF and VG). Interobserver agreement was assessed by a single-score intraclass correlation coefficient (ICC), which was calculated with the package irr. Agreement was considered high, good, moderate, and low when the ICC was > 0.90, between 0.75 and 0.90, between 0.50 and 0.75, and <0.50, respectively [31].

## 3. Results

### 3.1. Study Population

A total of 100 CKCSs were included in the study: 28 dogs in the control group (stage A) and 72 in the diseased group (21 in stage B1, 29 in stage B2, and 22 in stage C). In the diseased group 22/72 (30.6%) dogs were retrospectively enrolled (10/22 dogs from ADVETIA Veterinary Hospital and 12/22 dogs from Vetradmobile), and 50/72 (69.4%) dogs were prospectively recruited (48/50 dogs from ADVETIA Veterinary Hospital and 2/50 dogs from Médi-Vet Hospital). The retrospectively recruited cases were classified as follows: 3 dogs in stage B1 (3/21, 14.3%), 12 in stage B2 (12/29, 41.4%), and 7 in stage C (7/22, 31.8%).

The epidemiological and clinical characteristics of the control group and the three subgroups (B1, B2, and C) are shown in Table 1. The control group included a higher proportion of females (18/28, 64.3%), whereas the diseased group showed an approximately equal distribution of males (37/72, 51.4%) and females (35/72, 48.6%). However, no significant difference in gender ratio was observed among the groups (*p* = 0.3952).

Age differences were present among the groups A, B1, B2, and C (*p* < 0.001). Dogs in stage A (the control group) were younger than diseased dogs in stages B1, B2, and C (Table 1). Age was not significantly different between dogs in stage B2 and stage C (*p* = 0.518).

Body weight was significantly higher in stage B1 and stage B2 dogs than in dogs in the control group (*p* = 0.0023 and *p* = 0.0012, respectively). There was no significant difference in BW between the different diseased subgroups, namely B1, B2, and C.

Cardiovascular medications administered at the time of echocardiographic evaluation are summarized in Table 2. Regarding diuretic therapy in stage C dogs, 6/22 dogs had not received diuretics prior to the echocardiographic examination. One dog had been treated with diuretics for less than one week but more than 24 h, while the remaining fifteen dogs had been on diuretic therapy for more than two weeks at the time of echocardiography.

### 3.2. Variability of PV Diameter Measurements

Intraobserver CVs for PV1, PV2, and PV3 diameters were 3.4%, 2.8%, and 4.7%, respectively, for the first observer (VG) and 1.7%, 1.3%, and 2.6%, respectively, for the second observer (CF). Interobserver CVs for PV1, PV2, and PV3 diameters were, respectively, 2.7%, 2.1%, and 4.4%. Interobserver agreement was high (ICC > 0.90) for the three PV diameters, namely PV1, PV2, and PV3 (Table 3).

### 3.3. Pulmonary Vein Diameters and BW

Correlations between BW and PV1, PV2, and PV3 diameters were analyzed in the 28 dogs of the control group. The weakest correlation between BW and the PV diameter was observed for PV2 (rho-PV2 = 0.24, [*p* = 0.247]). Correlations between BW and PV1 or PV3 were higher (rho-PV1 = 0.51, [*p* = 0.007]; rho-PV3 = 0.36, [*p* = 0.062]). This result led us to only select PV2 for subsequent analysis.

### 3.4. Measurements of PV2 Diameter According to the ACVIM Stage and Correlations with Echocardiographic Parameters

**Figure 2:**
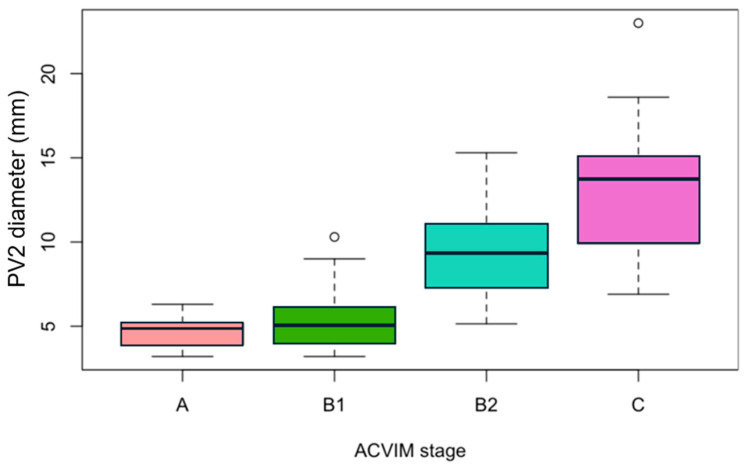
Boxplots showing pulmonary vein 2 diameters in 92 Cavalier King Charles Spaniels including groups of different stages of myxomatous mitral valve disease according to ACVIM classification (stages B1, B2, and C) and a control group (stage A). The central line within each box represents the median; the length of the box represents the interquartile range (25th–75th percentiles), and the whiskers indicate the 10th and 90th percentiles. Outliers are represented by empty circles.

The diameter of PV2 was measured in 26/28 dogs (92.9%) in stage A, 18/21 dogs (85.7%) in stage B1, 27/29 dogs (93.1%) in stage B2, and 21/22 dogs (95.5%) in stage C. The median (IQR) PV2 diameter was 4.9 mm (3.9–5.2) in stage A, 5.1 mm (4.0–6.0) in stage B1, 9.3 mm (7.3–11.1) in stage B2, and 13.7 mm (9.9–15.1) in stage C (Figure 2).

There was no significant difference in PV2 diameter between stages A and B1 (*p* = 0.242).

The diameter of PV2 was significantly higher in stage B2 dogs than in stage B1 dogs (*p* < 0.001) and in stage C dogs than in stage B2 dogs (*p* = 0.003).

Relevant key echocardiographic variables are summarized in Table 4. As expected, there was a significant global increase in the LVIDDN, the LA:Ao, the TR PG, the RF, the E, and the ratio of E to A with the progression from stage A to stage C.

**Figure 3:**
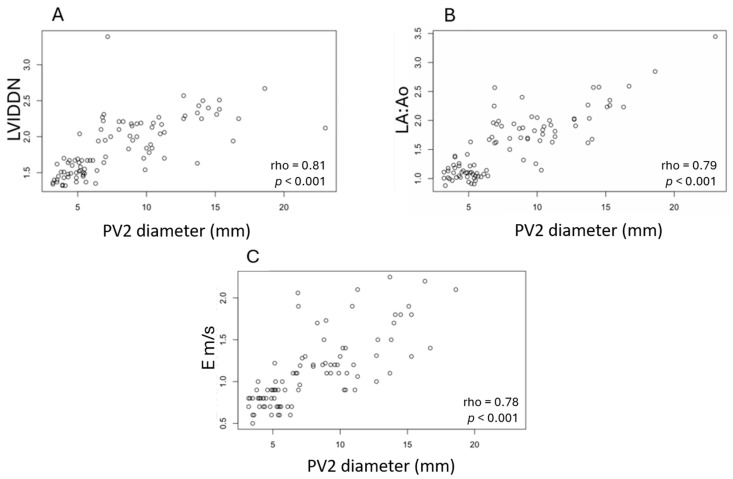
Significant correlations between pulmonary vein 2 diameter and (**A**) left ventricular internal diameter in diastole normalized for body weight, (**B**) ratio of the left atrial dimension to the aortic annulus dimension, and (**C**) peak velocity of early diastolic transmitral flow in 92 Cavalier King Charles Spaniels with different stages of myxomatous mitral valve disease according to ACVIM classification, including control dogs. PV2, pulmonary vein 2; LVIDDN, Left ventricular internal diameter in diastole normalized for body weight; LA:Ao, ratio of the left atrial dimension to the aortic annulus dimension; E, peak velocity of early diastolic transmitral flow.

A significant positive correlation was detected between the PV2 diameter and the LVIDDN (rho = 0.81, *p* < 0.001), the LA:Ao ratio (rho = 0.79, *p* < 0.001), the mitral inflow E wave peak velocity (rho = 0.78, *p* < 0.001) (Figure 3), the RF of mitral regurgitation (rho = 0.69, *p* < 0.001), and the TR PG (rho = 0.65, *p* < 0.001).

### 3.5. Radiographic Score and PV2 Diameter

Thoracic radiographs were available in all control dogs (28/28) and 63/72 dogs (87.5%) with MMVD. The two radiologists (HG and YR) scored the 28 radiographic studies in all 28 control dogs. Some radiographic studies in dogs with MMVD were considered of poor technical quality and excluded from the reading. Consequently, one radiologist scored radiographic studies in 55/63 dogs (87.3%) with MMVD and the other in 60/63 (95.2%).

The interreader agreement for radiographic scoring was moderate (ICC: 0.655, [95% CI, 0.186–0.835], and *p* = 0.004). For both readers a significant positive correlation was found between echocardiographic PV2 diameter and radiographic scores (rho-YR = 0.695, *p* < 0.001 and rho-HG = 0.789, *p* < 0.001). Additionally, for both readers, there was a significant association between the radiographic scores and the stages of MMVD, with a higher score indicating a more severe stage (*p* < 0.001).

### 3.6. Diameter of PV2 as a Diagnostic Marker for Differentiating Stages B1, B2, and C

The optimal cut-point value of PV2 diameter for discriminating stage B2 from stage B1 groups was 6.5 mm and was associated with a sensitivity of 96% (95% CI: 79–100%), a specificity of 83% (95% CI: 57–96%), and a positive predictive value of 85.2% (95% CI: 72–97%). The performance of the readers using PV2 for discriminating stage B2 from stage B1 groups was good (AUC, 0.916 and [95% CI: 0.82–1]).

**Figure 4:**
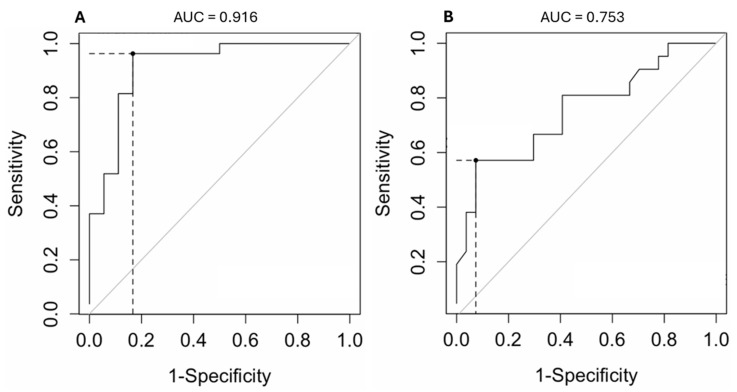
Receiver operating characteristic (ROC) curve (solid line) illustrating the performance (sensitivity and specificity) of pulmonary vein 2 optimal cut-off value (corresponding to the point on the ROC curve) for distinguishing stage B2 from B1 ((**A**): threshold = 6.5 mm) and stage C from B2 ((**B**): threshold = 12.8 mm) according to ACVIM classification. The dotted lines indicate on each ROC curve the diagnostic performance of the chosen cut-off value.

The optimal cut-point value of PV2 diameter for discriminating stage C from stage B2 groups was 12.8 mm and was associated with a sensitivity of 57% (95% CI: 34–77%), a specificity of 93% (95% CI: 74–99%), and a PPV of 88.5% (95% CI: 56–97%). The performance of the readers using PV2 for discriminating stage C from stage B2 groups was moderate (AUC, 0.753 and [95% CI: 0.61–0.90]) (Figure 4).

## 4. Discussion

This study described another access view to measure PV diameters in healthy CKCSs and CKCSs at different stages of MMVD. Statistical analysis confirmed the method to be repeatable and reproducible, which enhances its relevance as an additional biomarker of congestive status.

The study was focused on a single pulmonary vein, PV2, as PV2 was the least affected variable by BW compared with PV1 and PV3. According to statistics, PV1 and PV3 were not highly impacted by BW, suggesting that they could potentially serve as alternative diagnostic tools if PV2 is unavailable. Nevertheless, this hypothesis remains to be confirmed in future studies. Even so, as the central position of PV2 permits proper alignment with the ultrasound beam, PV2 is also the easiest PV to visualize and, hence, the most measurable PV, making its diameter measurement less prone to misregistration. From a practical standpoint, the left parasternal long axis view is part of a standard echocardiographic examination, and the additional measure of PV2 does not add too much time to the exam, which is valuable in dyspneic dogs. The timing of the measurement, which is taken at the end of systole, is crucial because similar to other veins, the PV size varies during the cardiac cycle [11]. By selecting the end-systolic phase, all veins were measured at the same time point. We selected the end of the T-wave, since the PV size is maximal at end atrial diastole, that is, just prior to the opening of the mitral valve [27]. Unsurprisingly, all operators felt that PV measures were easier to take from dogs in advanced stages of MMVD since the PVs and LA were distended. The corollary is that a smaller PV size in dogs from the control group may have led to the underestimation of PV1 and PV3 diameters, which are already more difficult to visualize.

The PV/PA ratio, previously described as an index of CHF in dogs with MMVD that increases with the severity of HF [14,32], may potentially be underestimated by post- or precapillary PH [15,16,17,18]. Our study focused exclusively on the diameter of the PVs on a single breed commonly affected by MMVD, the CKCS [1,21], with the aim to remove confounding effects. The single measure of PV is not affected by PH. According to Starling’s law, pulmonary edema develops when the hydrostatic pressure exceeds the normal plasma colloid osmotic pressure in PVs, disrupting the lung’s fluid balance. This imbalance leads to excessive interstitial fluid accumulation, surpassing the drainage capacity of the lymphatic system. Increased hydrostatic pressure is most commonly caused by elevated left ventricular filling pressures, leading to a rise in pulmonary venous and LA pressures [7,8]. Given the fundamental role of pulmonary venous pressure in the pathophysiology of pulmonary edema, our study focused on the PV diameters as an indirect sign of pulmonary venous hypertension, and thus it is a potential aid in identifying early signs of edema.

The PV diameter is used in people to guide timely diuretic therapy, potentially preventing the progression to overt, life-threatening pulmonary edema [9,10]. To our knowledge, there is no study evaluating the absolute echocardiographic PV diameter as a reliable biomarker of congestive status in dogs. A particularly relevant clinical application of PV diameter could be the evaluation of dyspneic dogs when thoracic radiographs are difficult to obtain and the underlying cause of respiratory distress is unclear, specifically when differentiating left-sided CHF from primary respiratory disease or other causes of dyspnea. This approach can be valuable in small breed dogs or those predisposed to MMVD, particularly when a loud mitral murmur is present, as the probability of left-sided CHF is inherently higher in this population. In such cases, the high positive predictive value of PV2 in discriminating stage C from stage B2 dogs (88.5%) and stage B2 from stage B1 dogs (85.2%) suggests a potential diagnostic utility of PV2 in supporting clinical decision-making.

Our study highlighted a significant increase in PV2 diameter in relation to disease progression, emphasizing the utility of PV echocardiographic measurements in assessing the severity of MMVD. The absence of a significant difference in PV2 diameter between stages A and B1 is consistent with the early clinical progression of MMVD, as mitral regurgitation in these stages is not yet severe enough to induce cardiac remodeling. Conversely, PV2 diameter showed a significant higher value in stage B2 compared with B1 and in stage C compared with B2. These changes reflect the escalating hemodynamic impact of MMVD as the disease progresses, with worsening mitral regurgitation leading to left atrial and ventricular dilation and elevated pulmonary venous pressures.

A significant positive correlation between PV2 diameter and some selected echocardiographic parameters was identified. Unsurprisingly, the strongest correlations were observed with the LVIDDN and the LA:Ao. A strong correlation was also found between the PV2 diameter and the mitral inflow E-wave peak velocity. This association most likely reflects the increase in left ventricular filling pressure that is expected in both pulmonary venous congestion/dilation and the increase in mitral inflow E-wave velocities in dogs with advanced MMVD. Our study identified a significant correlation between PV2 diameter and the RF that was moderate. As demonstrated by Kittleson et al., the RF calculated by the PISA method showed significant increases across disease stages and strong correlations with echocardiographic parameters, including LA size and LV internal diameter [25].

Our study found two cut-off values for the PV2 diameter in CKCS dogs. A cut-off value of 6.5 mm is proposed for distinguishing stages B2 and B1 with high sensitivity (96%) and specificity (83%). This distinction is likely attributable to the significant cardiac remodeling, particularly LA dilation, that occurs as the disease progresses from B1 to B2. The remodeling leads to more pronounced differences between normal and dilated left atria and, consequently, between normal and dilated PVs. In this context, it is also conceivable that LA dilation may lead to mechanical deformation and the stretching of the pulmonary vein ostia, not only as a result of increased pressure but also due to structural remodeling and atrial wall tension. A cut-off value of 12.8 mm is proposed for differentiating stages B2 and C with high specificity (93%) but low sensitivity (57%). The low sensitivity reflects the overlap in PV2 diameters between the B2 and C groups, as the PV may reach a plateau in dilation despite the worsening of mitral regurgitation and heart failure. While these findings are relevant to CKCSs, breed-related differences should be taken into account, and additional studies are necessary to assess their applicability to other breeds.

All animals in our study, including those in the control group, were also evaluated radiographically to confirm the presence or absence of pulmonary edema. The radiographic examination in the control group aimed to exclude animals with subclinical respiratory conditions that could affect pulmonary pressure and establish a reference score for healthy animals, allowing for a comparison with those affected by MMVD. A significant positive correlation was found between echocardiographic PV2 diameter and radiographic scores for both operators. The moderate agreement between radiologists pinpoints the subjective interpretation of some radiographic signs, particularly a mild interstitial unstructured lung pattern and a mild-to-moderate dilation of PVs.

This study has several limitations. Firstly, as this study was multicentric and partially retrospective (22% of the dogs were selected retrospectively), all echocardiographic examinations were not standardized, and the recorded images used for measuring PV2 were not obtained by a single operator. However, this limitation may have been tempered as all measurements of PV diameters were consensually taken by the same two readers. The high interobserver and intraobserver level of agreement in the PV diameter measurements led us to consider that the study design did not have a main impact on the results. In addition, some dogs in stage C were in a decompensated state of HF at the time of the echocardiographic examination, while others were in a compensated state. Ongoing treatments and emergency care treatments including diuretics (furosemide, torasemide, and spironolactone) and vasodilators (ACE inhibitors and pimobendan) probably had an impact and may have reduced the overall PV diameters. However, animal welfare was a priority, and dogs with severe clinical signs were first treated before the echocardiographic examination was performed. The small number of dogs in group C in decompensated CHF prevented direct comparisons between this subpopulation and dogs in stage B2. Lastly, several physiological and clinical parameters—such as volemic status, intrathoracic pressure variations, and body position—may potentially influence PV diameter measurements. Further investigation is needed to assess the extent of these effects.

## 5. Conclusions

In this study, the PV2 diameter appeared as a simple and reproducible echocardiographic parameter that correlated strongly with the progression of MMVD in CKCSs. This parameter may provide valuable insights for identifying CKCSs in the early stages of CHF. PV2 measurements could potentially support clinicians in the decision-making process, particularly regarding the initiation of diuretic therapy. However, given the modest sensitivity observed, its use should be considered complementary and interpreted alongside other clinical and echocardiographic findings.

## Figures and Tables

**Table 1:** Epidemiological data, clinical findings, and physical examination results in 72 Cavalier King Charles Spaniels with myxomatous mitral valve disease (stages B1, B2, and C) and 28 control dogs (stage A) classified according to the ACVIM consensus statement. Data are presented for each group as medians (and interquartile ranges: 25th–75th percentiles) for continuous data and the number of dogs (and percentage) for categorical data. For each continuous variable (age, weight, and HR), significant differences between groups are indicated by different superscript letters (a, b, or c): groups sharing common letters are not significantly different (Wilcoxon rank sum test with Bonferroni correction). HR, heart rate.

	A (n = 28)	B1 (n = 21)	B2 (n = 29)	C (n = 22)
Age (years, IQR)	2.47 (1.80–3.14) ^a^	6.41 (4.44–8.12) ^b^	8.84 (8.04–10.23) ^c^	9.65 (8.98–10.41) ^c^
Weight (kg, IQR)	7.60 (6.45–9.43) ^a^	9.00 (8.40–10.60) ^b^	9.80 (8.30–10.50) ^b^	8.70 (7.28–9.93) ^ab^
Males (ratio, %)	10/28 (35.7%)	10/21 (47.6%)	17/29 (58.6%)	10/22 (45.5%)
Cough (ratio, %)	-	1/21 (4.8%)	10/29 (34.5%)	8/22 (36.4%)
Exercise intolerance (ratio, %)	-	-	9/29 (31.0%)	5/22 (22.7%)
Tachypnea/Dyspnea (ratio, %)	-	-	3/29 (10.3%)	12/22 (54.5%)
Syncope (ratio, %)	-	-	2/29 (6.9%)	3/22 (13.6%)
Ascites (ratio, %)	-	-	-	2/22 (9.1%)
HR (beats/min, IQR)	128 (114–141) ^a^	130 (115–140) ^a^	140 (133–150) ^ab^	150 (141–155) ^b^
Murmur grade 0/6 to 6/6 (IQR)	-	3 (2–4)	4 (4–5)	5 (4–5)

**Table 2:** Cardiovascular medications administered at the time of echocardiographic assessment in 72 Cavalier King Charles Spaniels with myxomatous mitral valve disease classified according to the ACVIM consensus statement (stages B1, B2 and C). Data are presented for each group as the number of dogs (and percentage) treated with different drugs.

Drugs	B1 (n = 21)	B2 (n = 29)	C (n = 22)
Pimobendan	4 (19.0%)	20 (69.0%)	20 (90.9%)
Benazepril	2 (9.5%)	15 (51.7%)	11 (50.0%)
Spironolactone	1 (4.8%)	11 (37.9%)	13 (59.1%)
Furosemide	-	2 (6.9%)	15 (68.2%)
Torasemide	-	2 (6.9%)	9 (40.9%)
Sildenafil	-	1 (3.4%)	3 (13.6%)
Digoxin	-	-	1 (4.5%)

**Table 3:** Interobserver agreement analysis for the three pulmonary veins’ diameters assessed using the single-score ICC. All the measurements showed a high agreement value (ICC > 0.90). *p*, probability of the null hypothesis (ICC = 0).

	ICC	95% CI	*p*
Interobserver PV1 diameter	0.988	0.982–0.993	<0.001
Interobserver PV2 diameter	0.997	0.995–0.998	<0.001
Interobserver PV3 diameter	0.994	0.990–0.996	<0.001

**Table 4:** Echocardiographic variables in 72 Cavalier King Charles Spaniels with myxomatous mitral valve disease according to ACVIM stages and in 28 control dogs (stage A). Data are presented as medians (and interquartile ranges: 25th–75th percentiles). For each variable, *p* values correspond to global differences among groups assessed by Kruskal–Wallis rank sum tests. Significant differences between groups are indicated by superscript letters (a, b, c, or d): groups sharing common letters are not significantly different (Wilcoxon rank sum test with Bonferroni correction). LVIDDN, left ventricular internal diameter in diastole normalized for body weight; LA:Ao, ratio of the left atrial dimension to the aortic annulus dimension; TR, tricuspid regurgitation; PG, pressure gradient; RF, regurgitant fraction; E, peak velocity of early diastolic transmitral flow; and E:A, ratio of the peak velocity of early diastolic transmitral flow to the peak velocity of late diastolic transmitral flow. * available for *n* number of dogs.

Echocardiographic Variables	A	B1	B2	C	*p*
LVIDDN (cm/kg)	1.51 (1.42–1.61) ^a^ * 28/28 dogs	1.53 (1.47–1.67) ^a^ * 21/21 dogs	2.13 (2.00–2.22) ^b^ * 29/29 dogs	2.26 (1.98–2.38) ^b^ * 22/22 dogs	<0.001
LA:Ao	1.07 (1.00–1.13) ^a^ * 28/28 dog	1.18 (1.08–1.32) ^b^ * 21/21 dogs	1.81 (1.68–1.99) ^c^ * 29/29 dogs	2.14 (1.92–2.51) ^d^ * 22/22 dogs	<0.001
TR PG (mmHg)	16.0 (11.5–18.0) ^a^ * 19/28 dogs	26.5 (21.3–35.8) ^b^ * 18/21 dogs	50.0 (31.0–57.5) ^c^ * 27/29 dogs	63.0 (40.0–75.0) ^c^ * 21/22 dogs	<0.001
RF (%)	/	26.5 (13.0–36.5) ^a^ * 14/21 dogs	58.0 (51.0–63.0) ^b^ * 19/29 dogs	76.5 (65.5–79.8) ^c^ * 18/22 dogs	<0.001
E (m/s)	0.8 (0.7–0.9) ^a^ * 28/28 dogs	0.8 (0.7–0.9) ^a^ * 21/21 dogs	1.2 (1.1–1.4) ^b^ * 29/29 dogs	1.7 (1.2–1.9) ^c^ * 21/22 dogs	<0.001
E:A	1.29 (1.16–1.43) ^a^ * 28/28 dogs	1.17 (1.17–1.17) ^a^ * 21/21 dogs	1.33 (1.17–1.57) ^a^ * 28/29 dogs	2.00 (1.63–2.43) ^b^ * 21/22 dogs	<0.001

## Data Availability

The original contributions presented in this study are included in the article/Supplementary Materials. Further inquiries can be directed to the corresponding authors.

## Supplementary Materials

The following supporting information can be downloaded at https://www.mdpi.com/article/10.3390/vetsci12070615/s1, Figure S1. Correlations between pulmonary vein 2 diameter and (A) the tricuspid regurgitation pressure gradient, (B) the regurgitant fraction, and (C) the E:A ratio in Cavalier King Charles Spaniels with different stages of myxomatous mitral valve disease according to ACVIM classification, including control dogs. ACVIM, American College of Veterinary Internal Medicine; PV2, pulmonary vein 2; TR PG, tricuspid regurgitation pressure gradient; RF, regurgitant fraction; and E:A, ratio of E (peak velocity of early diastolic transmitral flow) to A (peak velocity of late diastolic transmitral flow).

## Abbreviations

The following abbreviations are used in this manuscript:

A	Peak velocity of late diastolic transmitral flow
ACVIM	American College of Veterinary Internal Medicine
AUC	Area under the ROC curve
BW	Body weight
CHF	Congestive heart failure
CI	Confidence interval
CKCSs	Cavalier King Charles Spaniels
CV	Coefficient of variation
E	Peak velocity of early diastolic transmitral flow
HF	Heart failure
ICC	Intraclass correlation coefficient
LA	Left atrium
LA:Ao	Ratio of the left atrial dimension to the aortic annulus dimension
LVIDDN	Left ventricular internal diameter in diastole normalized for body weight
LV	Left ventricle
MMVD	Myxomatous mitral valve disease
PISA	Proximal isovelocity surface area
PG	Pressure gradient
PH	Pulmonary hypertension
PPVs	Positive predictive values
PV	Pulmonary vein
PVs	Pulmonary veins
PV/PA	Pulmonary vein diameter-to-pulmonary artery diameter ratio
RF	Regurgitant fraction
ROC curve	Receiver operating characteristic curve
TR	Tricuspid regurgitation
VHS	Vertebral heart score (VHS)
VLAS	Vertebral left atrial size (VLAS)
