# Supplementary figures and images for: Assessment of Pulmonary Vein Diameters in Cavalier King Charles Spaniels with Myxomatous Mitral Valve Disease
Source: Vet Sci. 2025 Jun 24;12(7):615. doi: 10.3390/vetsci12070615 (PMC12299408; doi:10.3390/vetsci12070615)

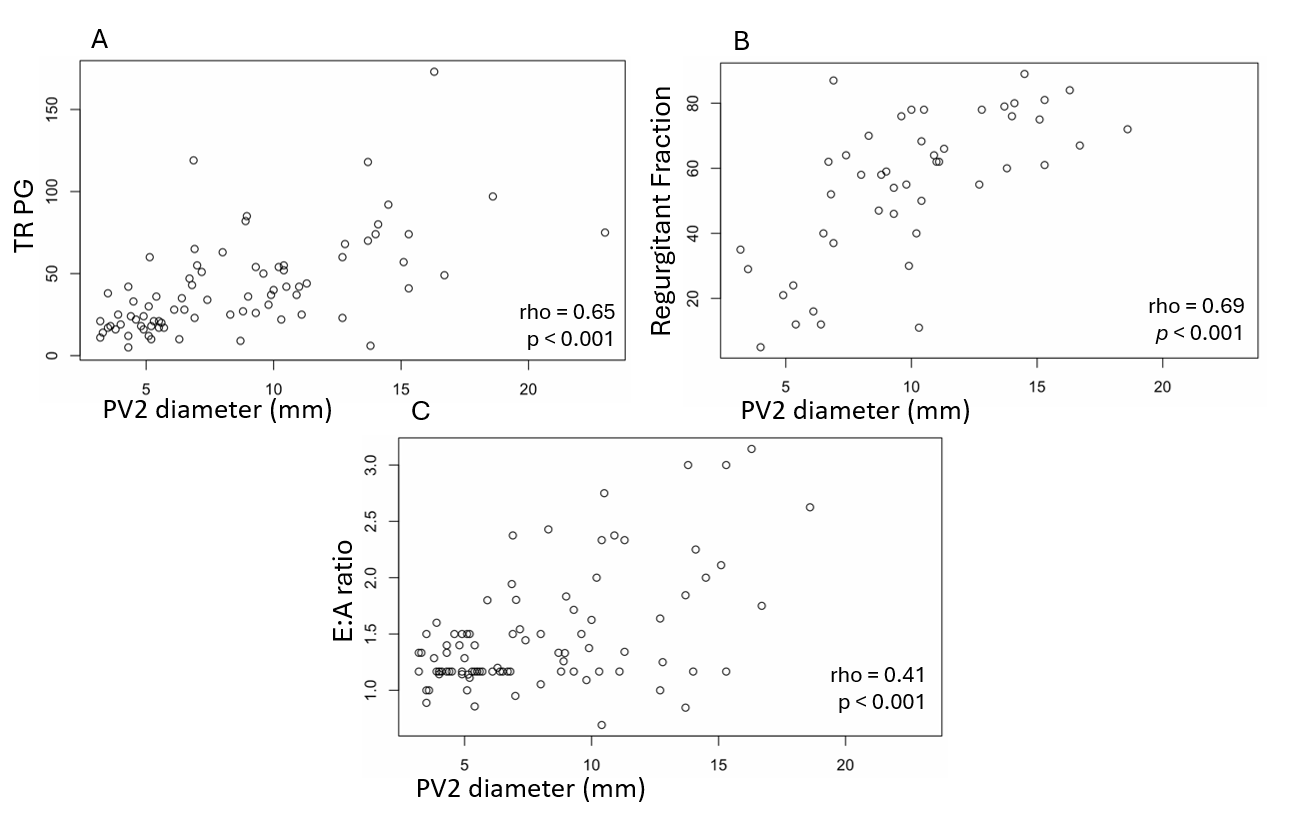

Supplement: Supplementary file 1 [file vetsci-12-00615-s001.zip › vetsci-3652967-supplementary.png]
